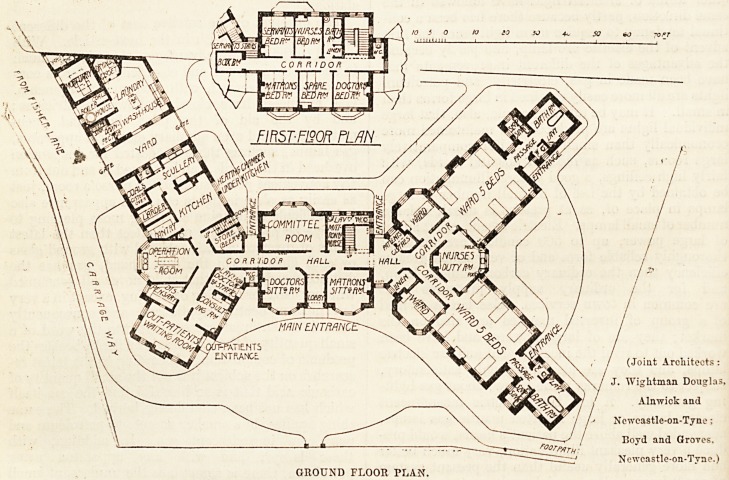# New Infirmary, Alnwick

**Published:** 1909-03-06

**Authors:** 


### NEW INFIRMARY, ALNWICK.

This building, opened by the Duke of Northumberland on Nov. 4, 1908, replaces the building which was erected in 1819 as a dispensary, and in 1849 changed into an infirmary. It was at first proposed to make extensive alterations in the old building, but on the advice of Mr. Leeson, of Messrs. Oliver and Leeson, architects, of Newcastle, the committee wisely decided to erect a new building rather than spend money in altering the old one.

Having obtained through the good offices of the Duke of Northumberland a suitable site on favourable terms, the committee advertised for plans, and, on the nomination of the President of the Royal Institute of British Architects, appointed Mr. Frank Caws, F.R.I.B.A., of Sunderland, to act as assessor. The result was that, out of 20 designs submitted, the plans by Mr. J. Wightman Douglas and Messrs. Boyd and Groves were placed first, and the work was entrusted to those gentlemen.

The plans, which we publish to-day, show a very ingeniously worked out scheme on a site that would appear to be somewhat cramped. The outline of the building takes, roughly, the form of the letter X, the four arms being one story in height, while the centre block is two stories. The points of the compass are unfortunately omitted from the plan, but we infer from the description in the local press that a line drawn through the centre of the operation-room along the corridor and through the centre of the nurses' duty room would lie approximately north and south. Thus the wards receive a maximum of sunlight.

The ward block is compact and well arranged; the nurses' duty-room separates the two large wards, and is equally conveniently placed for them and for the two separation wards with one bed each. The sanitary offices are adequate and properly cut off from the wards, and a door from each lobby into the open air affords access for patients to the garden, while at the same time it is a convenient mode of access for workmen to the sanitary offices for the purpose of repairs. The centre block contains, on the ground floor, sitting-rooms for medical officer and matron, committee room, and lavatory and w.c. for matron and nurses. The upper floor contains bedrooms for staff and servants.

In the northern wings are placed the out-patient waiting-hall, consulting-room with lavatory and w.c. for medical staff, dispensary, operation-room, and the kitchen offices. The position of the operation-room close to the out-patient department and the doctors' lavatory on one hand, and the kitchen offices on the other, is not a satisfactory arrangement; and the fact that a patient after operation has to be taken, on the way back to the ward, along a passage in which there are three doors to the external air certainly presents some element of danger. It would have been better if the operation-room had been placed nearer to the wards, and if some means had been taken to prevent contamination from the out-patient department. The position of the two staff w.c.'s inside the building is also unsatisfactory. In a small building such as this it is just as essential that the sanitary offices for the staff should be cut off as it is in the case of the ward offices.

The kitchen offices are well arranged and compact, but here again it would have been better if the entrance to the w.c. had been outside. The laundry, disinfection house, and mortuary form a separate building, and are well contrived. The arrangement for treating infected linen is unusually well thought out.

The exterior elevations are simply but effectively treated, and appropriately express the character of the building. The only detail open to criticism is the way in which the upper sashes are cut up by bars. Sash bars are out of place in a hospital, in that they provide so many unnecessary angles for dust; and from the architectural standpoint it is a mistake to divide up part of a window only.

(Joint Architects: J. Wightman Douglas, Alnwick and Newcastle-on-Tyne; Boyd and Groves, Newcastle-on-Tyne.)

GROUND FLOOR PLAN.

## Figures and Tables

**Figure f1:**